# Real-World Data of Tigecycline-Associated Drug-Induced Liver Injury Among Patients in China: A 3-year Retrospective Study as Assessed by the Updated RUCAM

**DOI:** 10.3389/fphar.2021.761167

**Published:** 2021-11-02

**Authors:** Xiaoping Shi, Chengchun Zuo, Lingling Yu, Donghui Lao, Xiaoyu Li, Qing Xu, Qianzhou Lv

**Affiliations:** ^1^ Department of Pharmacy, Zhongshan Hospital, Fudan University, Shanghai, China; ^2^ Department of Critical Care Medicine, Zhongshan Hospital, Fudan University, Shanghai, China

**Keywords:** tigecycline, liver injury, hepatotoxicity, causality assessment, RUCAM, roussel uclaf causality assessment method

## Abstract

**Background:** Tigecycline, a glycylcycline antibiotic, is increasingly used clinically for the treatment of severe infections caused by multidrug-resistant bacteria, but it is also associated with hepatotoxicity. However, the incidence and risk factors of tigecycline-associated drug-induced liver injury (DILI) are unclear. We conducted this study to investigate the incidence, characteristics and risk factors of tigecycline-associated DILI in the real-world clinic setting.

**Patients and Methods:** A retrospective analysis was conducted in inpatients who received tigecycline treatment from January 2018 to January 2020. Based on the biochemical criteria of DILI and the causality assessment by Roussel Uclaf Causality Assessment Method (RUCAM) using cases with a probable or highly probable causality grading, two clinical pharmacists and one clinician worked together to screen patients for tigecycline-associated DILI. Then patients with DILI were randomly matched by gender in a ratio of 1:2 to the remaining patients in the tigecycline cohort without biochemical abnormalities to identify risk factors.

**Results:** A total of 973 patients from 1,250 initial participants were included. The incidence of tigecycline-associated DILI was 5.7% (55/973). Among 55 DILI patients, 10 cases presented with the hepatocellular pattern, 4 cases belonged to the mixed pattern, and 41 presented with the cholestatic pattern. Most cases reached the severity of grade 1 and 2. The rate of recovery in hepatocellular pattern, mixed pattern, and cholestatic pattern was 70.0, 50.0, and 41.5%, respectively. The proportion of the DILI cases treated with high dose (100 mg) and prolonged duration (>14 days) was significantly higher than standard dose and routine duration (100.0% *vs.* 18.1%, *p* < 0.05). Logistic regression analysis showed that high maintenance dose (OR = 1.028, *p* = 0.002), prolonged duration (OR = 1.208, *p* = 0.000), and number of hepatotoxic drugs (OR = 2.232, *p* = 0.000) were independent factors of tigecycline-associated DILI.

**Conclusion:** Tigecycline was associated with liver injury, with a slightly higher incidence (5.7%) than the frequency of “frequent” (5%) defined by the Medical Dictionary for Regulatory Activities. Patients with a high maintenance dose and prolonged tigecycline regimen, as well as concomitant use of multiple hepatotoxic drugs should be paid more attention.

## Introduction

Tigecycline is the first clinically available glycylcycline antibiotic approved for the treatment of complicated intra-abdominal infection, complicated skin and soft-tissue infection, and community-acquired pneumonia, with a loading dose of 100 mg followed by 50 mg twice daily. It not only has good activity against Gram-positive and Gram-negative bacteria but also keeps highly sensitive to multidrug-resistant bacteria (Brink et al., 2010). Data from China Antimicrobial Surveillance Network (CHINET) showed that the resistance rates of *Acinetobacter* and *Enterobacter* to tigecycline were only 2.9% (http://www.chinets.com/). The expert consensus recommended tigecycline-based regimen as one of the options for the treatment of multidrug-resistant even pan-resistant Gram-negative bacteria (Bassetti et al., 2016; Guan et al., 2016), increasing its clinical application.

The most common adverse reactions of tigecycline are nausea and vomiting, but clinical trials in phase 2 and phase 3 found that tigecycline could cause elevated serum aminotransferase in 2–5% of patients (Ellis-Grosse et al., 2005; Oliva et al., 2005; Sacchidanand et al., 2005). The label of tigecycline provided by the manufacturer noted that isolated cases of severe liver dysfunction, cholestasis, and jaundice have been reported during post-marketing. Admittedly, both clinical trials and case reports have limitations. In particular, clinical trials are characterized by milder comorbidity, short drug duration, and relative homogeneity. It would compromise the understanding of the safety profile of tigecycline and could lead to insufficient attention to the potential hazards.

On the other hand, due to increasing bacterial resistance and limited available effective drug options, tigecycline was often prescribed off-label (Curcio et al., 2011; Moghnieh et al., 2017). The common off-label indications included ventilator-associated pneumonia, hospital-acquired pneumonia, and bacteremia etcetera. (Gardiner et al., 2010; Curcio et al., 2011; Moghnieh et al., 2017). In addition, based on the population pharmacokinetic and pharmacodynamic studies, the approved dose was often considered unable to achieve the optimal exposure in critically ill patients, thus the dosage of tigecycline was also off-label use (De Pascale et al., 2014; Xie et al., 2017). The prevalence of off-label use means that the adverse reactions might become more frequent and severe than those described in the label (Eguale et al., 2016).

So far, most current reports of tigecycline-associated liver injury were case reports or retrospective studies of small samples, and none had used a standardized, reliable method for assessing causality (Sabanis et al., 2015; Chen and Shi 2018; Geng et al., 2018; Xia and Jiang 2020). The purpose of this study was to investigate the incidence of tigecycline-associated drug-induced liver injury (DILI) in the real-world clinic setting by Roussel Uclaf Causality Assessment Method (RUCAM), describing the characteristics, management and outcomes, and exploring the risk factors of tigecycline-associated DILI.

## Methods

### Study Design and Patient Population

We conducted a single-center, retrospective study to investigate the incidence, characteristics, risk factors and outcomes of DILI in tigecycline-treated patients in the Zhongshan Hospital, Fudan University, with approximately 2,000 beds. Medical Ethics Committee of Zhongshan Hospital approved this study and waived the requirement for informed consent because this retrospective analysis was limited to preexisting data from medical records and collected as a part of the routine treatment by clinicians. Data extracted from electronic medical records were coded to ensure privacy issues confidentiality.

All adult inpatients (age ≥18 years) medicated with tigecycline between January 1, 2018 and January 1, 2020 were identified from the electronic medical records. The index date was the day of initiation of tigecycline during hospitalization. Patients were excluded if they met any of the following criteria: 1) incomplete laboratory data (lack of data obtained within 7 days prior to the index date or lack of follow-up liver function tests); 2) duration <72 h; 3) patients with Child C liver function.

**FIGURE 1:**
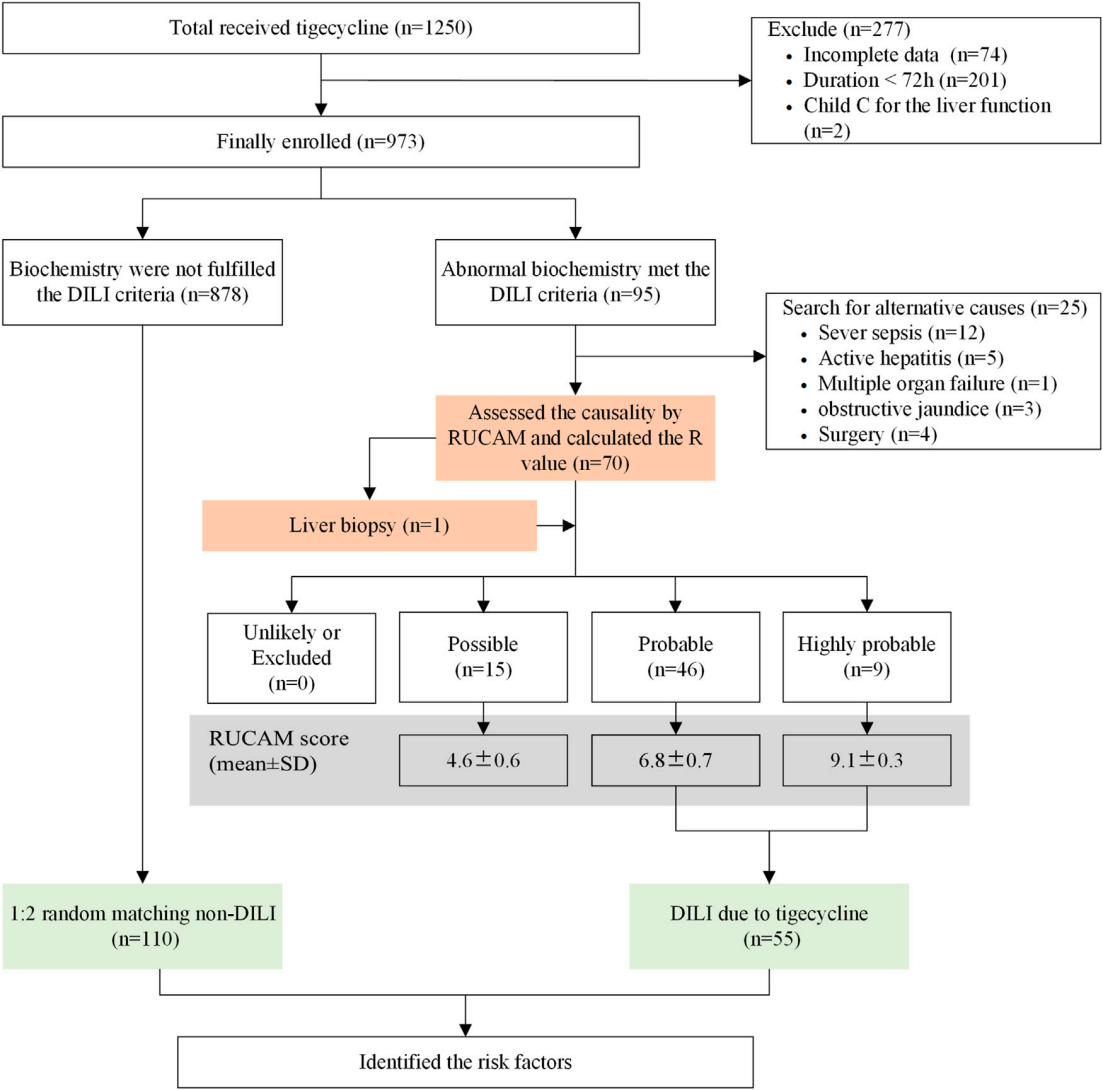
Study design. Abbreviation: DILI, drug-induced liver injury.

First, after the causality assessment by RUCAM (Danan and Teschke 2016), patients in DILI group were screened from eligible enrolled individuals. A flow chart summarizing the process of DILI case identification is presented in Figure 1. Second, in order to identify risk factors of tigecycline-associated liver injury, patients with DILI were randomly matched by gender in a ratio of 1:2 to the remaining patients in the tigecycline cohort without biochemical abnormalities.

### Data Collection

Demographic details and clinical information obtained from the electronic medical records were reviewed to identify the suspected DILI following tigecycline medication. Baseline values were defined as those obtained on the onset day of tigecycline or within 7 days prior to the onset date. The occurrence date of DILI was defined as the date after the initiation of tigecycline and within 3 days following discontinuation, in which the biochemical criteria of the DILI was reached. Patients were divided into two groups based on the results whether the level of liver enzyme elevations met the biochemical diagnostic criteria of DILI.

For patients whose elevated values of liver function fulfilling the biochemical diagnostic criteria of DILI, each record was reviewed in detail by two clinical pharmacists independently to determine the presence of suspected DILI. A standardized data collection form was applied for all eligible patients, including information regarding history of smoking or alcohol consumption, index date, diagnosis on admission, location of ward, history of liver disease, comorbidities, mechanical ventilation, tigecycline dose, duration of tigecycline therapy in days, baseline liver function and coagulation function, subsequently determined liver function and coagulation function, concomitant hepatotoxic medications, examinations for excluding other causes of liver injury (including hepatitis A virus (HAV), hepatitis B virus (HBV), hepatitis C virus (HCV), hepatitis E virus (HEV), Epstein-Barr virus (EBV), cytomegalovirus (CMV), herpes virus, Wilson’s disease, and autoimmune hepatitis) and clinical outcome. Indicators of liver function included alanine aminotransferase (ALT), aspartate aminotransferase (AST), alkaline phosphatase (ALP), gamma-glutamyltransferase (GGT), total bilirubin (TB), albumin (ALB). Indicators of coagulation function included prothrombin time (PT) and international normalized ratio (INR). If a liver-associated adverse event was listed as common one (incidence ≥1%) in the drug label of a suspected case concomitant drug, the drug was defined as a concomitant hepatotoxic medication.

For each DILI case, follow-up information on characteristics of DILI, treatment options, response of re-exposure, clinical outcomes and liver biopsy were collected if applicable. Characteristics of DILI included liver injury pattern and grade of severity, latency time of liver injury. Treatment options included withdrawal of tigecycline after liver injury, reduction of tigecycline dosage or continuous treatment without adjustment, and medical therapy for DILI. Outcomes of DILI included liver outcome categorized by recovery, improvement, no improvement, and aggravation, 30-day all-cause mortality, and length of stay in hospital.

### Biochemical Diagnostic Criteria of Suspected DILI Cases

Cases whose elevated liver enzymes met the following criteria were defined as the suspected DILI cases (Aithal et al., 2011; The Study of Drug Induced Liver Disease of Chinese 2015): 1) ALT ≥5×upper limit of normal (ULN), 2) ALT ≥3×ULN and TB ≥ 2×ULN, or 3) ALP ≥2×ULN, particularly with accompanying elevations in concentrations of GGT in the absence of known bone pathology driving the rise in ALP level. In patients with abnormal liver tests prior to starting treatment with tigecycline, ULN was replaced by the mean baseline values obtained before the index date of tigecycline and elevations should be calculated proportionate to this modified baseline.

### Causality Assessment of Suspected DILI

First, suspected DILI cases with alternative causes for the liver injury were excluded, including severe sepsis, active hepatitis, multiple organ failure, obstructive jaundice, and surgery. Then causality assessment of suspected DILI was conducted based on updated RUCAM (Danan and Teschke, 2016). The final score calculated from RUCAM led to the causality levels as follows: 0 points, excluded causality; 1-2, unlikely; 3-5, possible; 6-8, probable; and 9, highly probable. After the independent causality assessments, all suspected DILI cases were reviewed in detail by consensus of two clinical pharmacists and one clinician to ensure agreement on all assessments.

After ruling out alternative explanations for abnormal liver biochemical indicators and assessment by RUCAM, only patients with a RUCAM score ≥6 were stratified into the DILI group. Cases with a “possible” grading were excluded to minimize the influence on the evaluation of DILI, as this group showed a relatively weaker causal relationship between tigecycline and liver injury.

### Clinical Classification of DILI Patterns

Based on the Chinese guideline for the management of DILI (The Study of Drug Induced Liver Disease of Chinese 2015), we categorized the patients with DILI into 3 patterns on the basis of the ratio (R): 1) hepatocellular injury, ALT ≥3×ULN and R ≥ 5; 2) cholestatic injury, ALP ≥2×ULN and R ≤ 2; 3) mixed injury, ALT ≥3×ULN, ALP ≥2×ULN and 2 < R < 5. The R value was calculated for each DILI case on the day of the peak elevation of biochemical value which met the criteria of DILI. R was calculated as follows [(ALT _current_/ALT _baseline_)/(ALP _current_/ALP _baseline_)].

### Classification Criteria for the Severity of DILI

The severity of DILI was graded into five levels (The Study of Drug Induced Liver Disease of Chinese 2015):

Grade 1 (mild liver injury): The patients’ serum level of ALT or ALP is elevated, but total bilirubin (TB) < 2.5×ULN (42.75 μmol/L), and with INR <1.5;

Grade 2 (moderate liver injury): Patients with elevated serum levels of ALT or ALP, and TB ≥ 2.5×ULN or INR ≥1.5;

Grade 3 (severe liver injury): Patients with elevated serum levels of ALT or ALP, TB ≥ 5×ULN (85.5 μmol/L), with or without INR ≥1.5;

Grade 4 (acute liver failure): Evidence of coagulation abnormality indicated by INR ≥2 or PTA (prothrombin activity) < 40%, and TB ≥ 10×ULN (171 mmol/L) or daily increase ≥17.1 mmol/L;

Grade 5 (fatal): Death due to DILI or necessitates a liver transplant for survival.

### Definition on the Prognosis of DILI

Definition of the prognosis of DILI varied among different injury patterns. For hepatocellular injury, recovery was defined as ALT decreasing to below the ULN or baseline value; improvement was defined as ALT decreasing to below 3×ULN or baseline value; no improvement was defined as ALT not decreasing to below 3×ULN or baseline value; and aggravation was defined as ALT beyond the peak value.

For mixed injury, recovery was defined as both ALT and TB decreasing to below the ULN or baseline value; improvement was defined as ALT decreasing to below 3×ULN or baseline value, as well as TB decreasing to below; 2×ULN or baseline value; no improvement was defined as ALT not decreasing to below 3×ULN or baseline value, or TB not decreasing to below 2×ULN or baseline value; and aggravation was defined as either ALT or TB beyond the peak value.

For cholestatic injury, recovery was defined as ALP decreasing to below the ULN or baseline value; improvement was defined as ALP decreasing to below 2×ULN or baseline value; no improvement was defined as ALP not decreasing to below 3×ULN or baseline value; and aggravation was defined as ALP beyond the peak value. Both recovery and improvement of liver enzyme values can be regarded as effective outcomes.

### Statistical Analysis

Statistical analysis was performed through SPSS Statistics v.22.0 (IBM Corp., Armonk, NY, United States). Normally and non-normally distributed continuous variables were presented as mean ± standard deviations (SD) or median (interquartile ranges, IQR) and were compared by independent sample t-test and Mann-Whitney U test, respectively. Categorical variables were compared by Chi-square test or Fisher’s exact test. Variables with *p* value less than 0.1 in the univariate analysis were analyzed in Logistic regression model. A backward logistic regression model was adopted to analyze the independent risk factors of tigecycline-associated DILI. A *p* value <0.05 was considered statistically significant.

## Results

### Enrollment Process

A total of 1,250 patients treated-tigecycline were identified from the electronic medical records. 277 patients were excluded due to incomplete laboratory data (*n* = 74, 26.7%), duration of tigecycline less than 72 h (*n* = 201, 72.5%), and patients with Child C liver function (*n* = 2, 0.7%). Then, 95 of the assessed 973 patients had abnormal biochemical values. Among whom, 25 patients’ abnormal liver function could be explained by alternative causes, including severe sepsis (*n* = 12), active hepatitis (*n* = 5), multiple organ failure (*n* = 1), obstructive jaundice (*n* = 3), surgery (*n* = 4). Therefore, 70 patients were finally assessed by RUCAM and the R value (Figure 1).

### Clinical Outcome of DILI Pattern, Severity, and Treatment

Characteristics and outcomes of DILI caused by tigecycline were listed in Table1. Only one patient had performed liver biopsy. Based on the causality assessment by RUCAM, 15 cases with RUCAM scores between 3-5 were considered as possible, 46 cases whose RUCAM scores between 6-8 were considered as probable, and 9 cases with RUCAM scores equal to or beyond nine were considered as highly probable. After ruling out 15 cases with possible causality grade, 55 patients had confirmed tigecycline-associated DILI, with an incidence of 5.7% (55/973).

**TABLE 1:** Characteristics and outcomes of DILI caused by tigecycline.

Variables	Pattern of liver injury		
	Hep (*n* = 10)	Mix (*n* = 4)	Chol (*n* = 41)
Causality assessment			
Highly probable, *n* (%)	3 (30.0%)	0 (0.0%)	6 (14.6%)
Probable, *n* (%)	7 (70.0%)	4 (100.0%)	35 (85.4%)
Liver injury during tigecycline treatment, *n* (%)	7 (70.0%)	3 (75.0%)	32 (78.0%)
Withdrawal tigecycline after liver injury, *n* (%)	3 (30.0%)	2 (50.0%)	20 (48.8%)
Reduction tigecycline dosage after liver injury, *n* (%)	0 (0.0%)	0 (0.0%)	3 (7.3%)
Continuous treatment without adjustment, *n* (%)	4 (40.0%)	1 (25.0%)	9 (22.0%)
Liver injury within 3 days of discontinuation of tigecycline, *n* (%)	3 (30.0%)	1 (25.0%)	9 (22.0%)
Latency time of liver injury, median (IQR), days	4.5 (2.0–7.4)	6.5 (1.8–12.0)	12.0 (9.0–16.0)
Re-exposure to tigecycline and recurrent ALT or ALP increase, *n* (%)	0 (0.0%)	0 (0.0%)	2 (4.9%)
Drugs for treatment			
Anti-inflammation, *n* (%)	5 (50.0%)	2 (50.0%)	16 (39.0%)
Antioxidants, *n* (%)	8 (80.0%)	3 (75.0%)	31 (75.6%)
Phospholipids, *n* (%)	3 (30.0%)	2 (50.0%)	4 (9.8%)
Cholagogue, *n* (%)	3 (30.0%)	2 (50.0%)	19 (46.3%)
Outcome of liver injury*			
Recovery, *n* (%)	7 (70.0%)	2 (50.0%)	17 (41.5%)
Improvement, *n* (%)	1 (10.0%)	1 (25.0%)	5 (12.2%)
No improvement, *n* (%)	1 (10.0%)	0 (0.0%)	15 (36.6%)
Aggravation, *n* (%)	0 (0.0%)	1 (25.0%)	0 (0.0%)
Time to recovery, median (range, min-max), days	11.0 (4.0–37.0)	13.5 (2.0–25.0)	24.0 (8.0–66.0)
30-day all-cause mortality, *n* (%)	2 (20.0%)	1 (25.0%)	3 (7.3%)
Length of stay in hospital, median (IQR), days	42.0 (20.3–59.3)	33.5 (24.0–62.5)	46.0 (38.0–63.5)

* One of patients with hepatocellular injury pattern lack of outcome data, while eight of patients with cholestatic injury pattern. DILI, drug-induced liver injury; Hep, hepatocellular injury pattern; Mix, mixed injury pattern; Chol, cholestatic injury pattern; IQR, interquartile ranges; ALT, alanin aminotransferase; ALP, alkalinephosphatase; min, minimal; max, maximal.

According to the R value at initial valuation, 10 out of 55 DILI patients presented with the hepatocellular pattern, 4 cases belonged to the mixed pattern, and 41 presented with the cholestatic pattern (Table 1). When comes to initial evaluation on the grade of severity (Table 2), most cases among three patterns reached grade 1 and 2. It should be noticed that 5 cases in the cholestatic and 1 case in the mixed group reached grade 3, while 1 case in the cholestatic pattern was observed achieving grade 4.

**TABLE 2:** Characteristics and outcomes in DILI patients and non-DILI patients.

Severity of DILI	Hep		Mix		Chol	
	Initial valuation (*n* = 10)	Revaluation (*n* = 2)	Initial valuation (*n* = 4)	Revaluation (*n* = 2)	Initial valuation (*n* = 41)	Revaluation (*n* = 21)
Stage 1	5 (50.0%)	1 (50.0%)	2 (50.0%)	1 (50.0%)	25 (61.0%)	11 (52.4%)
Stage 2	5 (50.0%)	1 (50.0%)	1 (25.0%)	1 (50.0%)	10 (24.4%)	2 (9.5%)
Stage 3	0 (0.0%)	0 (0.0%)	1 (25.0%)	0 (0.0%)	5 (12.2%)	5 (23.8%)
Stage 4	0 (0.0%)	0 (0.0%)	0 (0.0%)	0 (0.0%)	1 (2.4%)	3 (14.3%)

DILI, drug-induced liver injury; Hep, hepatocellular injury pattern; Mix, mixed injury pattern; Chol, cholestatic injury pattern.

Only cases with continued deterioration of liver enzyme values were re-evaluated (Table 2). In one of the re-evaluated patients, the pattern of DILI changed from hepatocellular to mixed type on day 2 after discontinuation of tigecycline, and the severity grade increased from 1 to 2. Two patients changed from hepatocellular injury to cholestatic injury, one of them increased from level 2 to 3 on day 3 after withdrawal, and the other remained level 1 during the course. For mixed injury, no change in a liver injury pattern and severity level was observed. In addition, cases with cholestatic patterns changed only in the grade of severity. Specifically, severity developed from level 1 to level 2 was found in 1 case with cholestatic injury on the second day after withdrawal, 2 cases progressed from level 1 to level 3 on the last day of tigecycline treatment or the third day after withdrawal, 1 case progressed from level 1 to level 4 on the second day after withdrawal, 2 cases progressed from level 2 to level 3 during the course or the third day after withdrawal, 1 case progressed from level 2 to level 4 during the course, and 1 case progressed from level 3 to level 4 on the day after withdrawal.

For hepatocellular injury, mixed injury and cholestatic injury, the median latency of liver injury was 4.5 days (range, 2.0–7.4 days), 6.5 days (range, 1.8–12.0 days), and 12.0 days (range, 9.0–16.0 days), respectively (Table 1). Most patients developed DILI during tigecycline treatment, but 3 (30.0%) patients in the hepatocellular group, 1 (25.0%) in the mixed group and 9 (22.2%) in the cholestatic group developed DILI within 3 days of discontinuation of tigecycline. Recurrent double ALP increase as diagnostic criterion (Danan and Teschke 2016) was observed in two cholestatic cases when they were unintentionally re-challenged with tigecycline. No patient with other injury patterns underwent re-exposure to tigecycline in the study.

For patients with DILI during tigecycline treatment, approximately half of the patients classified in mixed (50.0%) and cholestatic pattern (48.8%) withdrew tigecycline after DILI onset, while 40.0% of patients in the hepatocellular group continued treatment without adjustment. Of the 55 patients with DILI, 50 cases received hepatoprotective drugs, antioxidants were most frequently administrated to treat DILI, followed by anti-inflammation agents, cholagogue, and polyene phosphatidylcholine (Table 1).

Notably, one of the patients with hepatocellular injury pattern and eight of patients with cholestatic injury pattern were lacking of data on DILI outcome. For the remaining parts (Table 1), patients with hepatocellular pattern had the highest rate of recovery (70.0%) and improvement (10.0%), followed by mixed pattern (50.0% for recovery and 25.0% for improvement), and then cholestatic pattern (41.5% for recovery and 12.2% for improvement). The median time to recovery for hepatocellular injury pattern was 11.0 days, 13.5 days for mixed pattern and 24.0 days for cholestatic pattern, respectively. Cholestatic cases seemed to have the longest median hospitalization length (46.0 days, IQR:38.0–63.5d), followed by hepatocellular cases (42.0 days, IQR:20.3–59.3d) and mixed cases (33.5 days, IQR:24.0–62.5d).

After excluding auto-discharged patients, 24 patients died within 30-day after being treated with tigecycline in the whole cohort. The 30-day all-cause mortality rate in the DILI group was 10.9% (6/55), with 2 cases in the hepatocellular type, 1 case in the mixed type and 3 cases in the cholestatic type. Among all types, three patients died from multiple systemic organ failure, two patients died from severe pneumonia, and the remaining patient died of malignant arrhythmia. As for the non-DILI group, the 30-day all-cause mortality rate was 16.4% (18/110), with seven patients died due to multiple systemic organ failure, five patients due to severe pneumonia, and the remaining six patients due to primary diseases or complications, including acute respiratory distress syndrome, gastrointestinal hemorrhage, heart failure, or NK/T-cell Lymphoma.

### Comparison of Biochemical Tests Between Baseline and After Tigecycline Treatment

**FIGURE 2:**
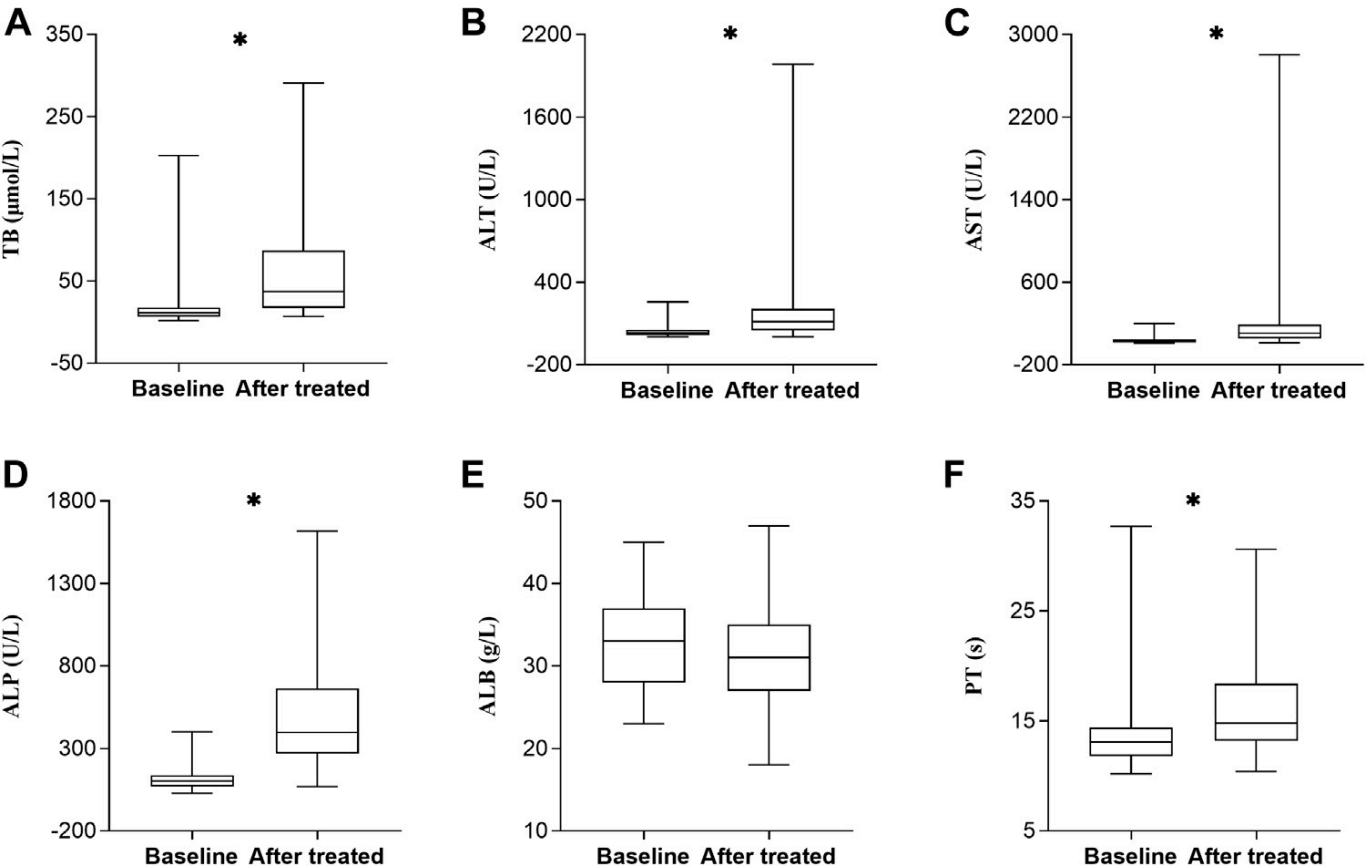
Changes of laboratory test values before and after tigecycline treatment in DILI patients (*n* = 55). Horizontal bars represent the median value, boxes represent the interquartile range and whiskers indicate the minimum and maximum value. Wilcoxon’s test was used to compared baseline and after tigecycline treatment’s biochemical test values. *p < 0.0001. Abbreviation: TB, total bilirubin; ALT, alanine aminotransferase; AST, aspartate aminotransferase; ALP, alkaline phosphatase; ALB, albumin; PT, prothrombin time; DILI, drug-induced liver injury.

As shown in Figure 2, we analyzed changes of biochemical tests of 55 patients with DILI before and after tigecycline treatment, to investigate whether tigecycline affected other liver function parameters to some extent. Our study found that not only ALT and ALP, but also median values of TB, AST, and PT were significantly increased after tigecycline treatment compared to baseline values in DILI cases (*p* < 0.0001).

### Analysis of Risk Factor Between DILI Group and Non-DILI Group

**FIGURE 3:**
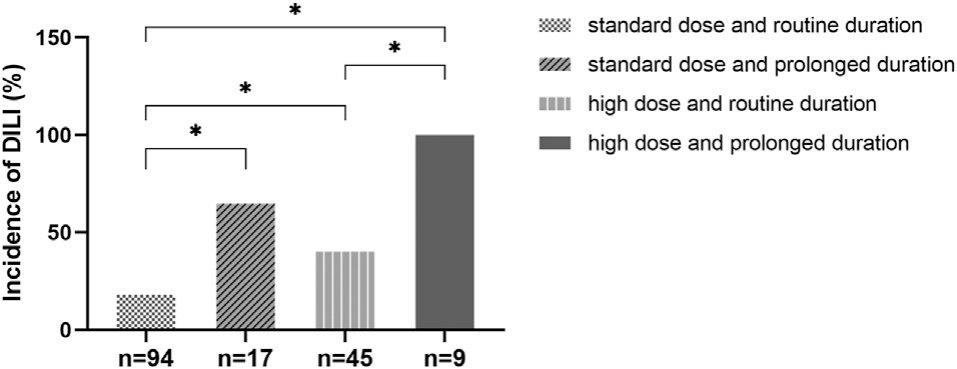
Effects of tigecycline treatment dose and duration on DILI. The proportion of the DILI cases treated with standard dose and routine duration, standard dose and prolonged duration, high dose and routine duration, high dose and prolonged duration was 18.1, 64.7, 40.0, and 100.0%, respectively. Abbreviation: DILI, drug-induced liver injury. *p < 0.05.

Characteristics of DILI group and non-DILI group were depicted in Table 3. 55 DILI patients were matched with 110 non-DILI patients. By univariate analysis, there was significant difference regarding mechanical ventilation, maintenance dose of tigecycline, duration of tigecycline and number of concomitant hepatotoxic medications between DILI group and non-DILI group. DILI patients with mechanical ventilation (58.2% *vs.* 29.1%, *p* = 0.000) were more likely to develop DILI. Meanwhile, proportion of patients with DILI among various maintenance doses or duration was summarized in Figure 3 and Table 3. Both high maintenance dose (100 mg) and prolonged duration (>14 days) of tigecycline were positively related to DILI risk. Proportion of the DILI cases treated with standard dose/prolonged duration was significantly higher than those treated with standard dose/routine duration (64.7% [11/17] *vs.* 18.1% [17/94], *p* < 0.05). Similar trends could be observed between high dose/routine duration and standard dose/routine duration (40.0% [18/45] *vs.* 18.1% [17/94], *p* < 0.05), high dose/prolonged duration and high dose/routine duration (100.0% [9/9] *vs.* 40.0% [18/45], *p* < 0.05), high dose/prolonged duration and standard dose/routine duration 100.0% [9/9] *vs.* 18.1% [17/94], *p* < 0.05), respectively (Figure 3). However, there showed no significant difference in terms of age, gender, weight, BMI, history of alcohol and smoke consumption, department of ward, history of liver disease, payments method, comorbidities, baseline laboratory variables, baseline liver function, loading dose of tigecycline, and 30-day all-cause mortality between two groups. Five variables (*p* < 0.1) were included in multivariate logistic regression analysis, including chronic liver disease, mechanical ventilation, maintenance dose, duration and number of concomitant hepatotoxic drugs. High maintenance dose (OR = 1.028, *p* = 0.002), prolonged duration (OR = 1.208, *p* = 0.000), and number of hepatotoxic drugs (OR = 2.232, *p* = 0.000) were found to be independent factors of tigecycline-associated DILI (Table 4).

**TABLE 3:** Characteristics and outcomes in DILI patients and non-DILI patients.

Variable	DILI (*n* = 55)	Non-DILI (*n* = 110)	*p*-value
Age, median (IQR), y	68 (55–75)	66 (57–77)	0.700
≤65 years, *n* (%)	23 (41.8%)	51 (46.4%)	0.580
>65 years, *n* (%)	32 (58.2%)	59 (53.6%)	
Male sex, *n* (%)	34 (61.8%)	68 (61.8%)	1.000
Weight, median (IQR), kg	60.0 (55.0–70.0)	62.0 (54.8–70.0)	0.974
BMI, median (IQR), kg/m^2^	22.7 (20.3–24.8)	22.7 (20.0–24.5)	0.753
<18.5 kg/m^2^, *n* (%)	5 (9.1%)	16 (14.6%)	0.589
18.5–24 kg/m^2^, *n* (%)	32 (58.2%)	58 (52.7%)	
>24 kg/m^2^, *n* (%)	18 (32.7%)	36 (32.7%)	
Hospital admission, *n* (%)			0.318
Medical ward	34 (61.8%)	59 (53.6%)	
Surgical ward	21 (38.2%)	51 (46.4%)	
Payment methods, *n* (%)			0.420
Medical insurance	38 (69.1%)	69 (62.7%)	
Self-paying	17 (30.9%)	41 (37.3%)	
Underlying disease, *n* (%)			
Chronic liver disease	6 (10.9%)	24 (21.8%)	0.087
Diabetes mellitus	13 (23.6%)	34 (30.9%)	0.329
Solid organ cancer	15 (27.3%)	36 (32.7%)	0.475
Hematologic malignancy tumor	0 (0.0%)	8 (7.3%)	0.096*
Heart disease	13 (23.6%)	38 (34.5%)	0.153
Smoking, *n* (%)	8 (14.5%)	18 (16.4%)	0.763
Alcohol use, *n* (%)	6 (10.9%)	9 (8.2%)	0.566
Mechanical ventilation, *n* (%)	32 (58.2%)	32 (29.1%)	**0.000**
Baseline laboratory variables			
ALB [40–55 g/L], median (IQR), g/L	33.0 (28.0–37.0)	31.5 (27.0–34.3)	0.108
PT [10.0–13.0 s], median (IQR), s	13.1 (11.8–14.4)	13.4 (12.0–14.5)	0.647
TB [3.4–20.4 μmol/L], median (IQR), μmol/L	11.5 (6.8–17.7)	12.2 (8.7–17.3)	0.295
ALT [Male: 9–50 U/L; Female: 7–40 U/L], median (IQR), U/L	33.0 (14.0–52.0)	22.0 (14.0–45.5)	0.219
AST [Male: 15–40 U/L; Female: 13–35 U/L], median (IQR), U/L	28.0 (17.0–44.0)	26.0 (17.0–43.5)	0.912
ALP [Male: 45–125 U/L; Female: age <50 years 35–100 U/L, age ≥50 years 50–135 U/L], median (IQR), U/L	102.0 (68.0–137.0)	91.0 (65.8–128.5)	0.674
GGT [Male 1060 U/L; Female 745 U/L], median (IQR), U/L	57.0 (27.0–119.0)	50.5 (27.8–107.8)	0.667
Baseline liver function			0.911
Normal, *n* (%)	22 (40.0%)	45 (40.9%)	
Abnormal, *n* (%)	33 (60.0%)	65 (59.1%)	
Tigecycline therapy			
Loading dose, *n* (%)	29 (52.7%)	64 (58.2%)	0.505
Maintaining dose, *n* (%)			**0.002**
Standard dose (50 mg)	28 (50.9%)	83 (75.5%)	
High dose (100 mg)	27 (49.1%)	27 (24.5%)	
Duration, median (IQR), days	13.0 (7.5–19.5)	7.0 (4.5–9.6)	**0.000**
≤14d, *n* (%)	35 (63.6%)	104 (94.5%)	
>14d, *n* (%)	20 (36.4%)	6 (5.5%)	
Number of concomitant hepatotoxic medications, median (IQR)	2.0 (1.0–4.0)	1.0 (1.0–2.0)	**0.000**
30-day all-cause mortality, *n* (%)	6 (10.9%)	18 (16.4%)	0.349

* Continuity correction. DILI, drug-induced liver injury; BMI, Body Mass Index; ALB: albumin; PT, prothrombin time; TB, total bilirubin; ALT, alanine aminotransferase; AST, aspartate aminotransferase; ALP, alkaline phosphatase; GGT, gamma-glutamyltransferase; IQR, interquartile ranges. The bold vaules provided in Table refers to *p*<0.05.

**TABLE 4:** Risk factors for DILI caused by tigecycline.

	β	S.E.	Wald χ^2^	Or (95%CI)	*P*
Chronic liver disease	0.594	0.616	0.928	1.811 (0.541–6.058)	0.335
Mechanical ventilation	−0.820	0.435	3.550	0.440 (0.188–1.033)	0.060
Maintaining dose	0.027	0.009	9.455	1.028 (1.010–1.046)	**0.002**
Duration	0.189	0.045	17.718	1.208 (1.106–1.319)	**0.000**
Number of concomitant hepatotoxic	0.803	0.216	13.771	2.232 (1.461–3.411)	**0.000**

DILI, drug-induced liver injury. The bold vaules provided in Table refers to *p*<0.05.

## Discussion

To the best of our knowledge, this single-center retrospective study involving 973 patients treated with tigecycline is the first to elucidate the incidence, characteristics, prognosis, and risk factors of tigecycline-associated DILI in China. The study provides real-world evidence for the hepatic safety profile in patients treated with tigecycline and has a relatively large sample size.

Off-label use (including high dosage) of tigecycline had been widely applied for critically ill patients, including patients with sepsis or septic shock (Curcio 2009; Conde-Estévez et al., 2010; Borsuk-De Moor et al., 2018). An early review displayed that high doses of tigecycline for patients were safe and tolerable (Peterson 2008). Regardless of some treatment-emergent adverse events, it seemed to be pretty safe.

Diagnosis of DILI is always considered a challenging issue due to non-specific symptoms and a lack of a valid diagnostic biomarker, often confounded by alternative causes. Therefore, identification of DILI requires exclusive diagnosis, and causality assessment by RUCAM might contribute to clarify the causality of the suspected DILI cases (Teschke and Danan 2016). At present, the diagnosis of DILI caused by tigecycline was very rare, and there was no literature on tigecycline-associated DILI (LiverTox, 2012). Some published studies on liver toxicity caused by tigecycline only mentioned the elevation of some liver enzyme levels, not classified by the biochemical criteria of DILI (Yahav et al., 2011; Kadoyama et al., 2012). Moreover, many DILI cases presented in LiverTox database were insufficiently documented without using RUCAM (Teschke and Danan 2021). There were gaps in the knowledge regarding the incidence, duration, pattern, and prognosis of tigecycline-associated DILI.

Herein, one of our key findings was that the incidence of tigecycline-associated DILI was 5.7% (55/973) in the real-world clinic setting, which appeared to be a little higher than the reported rate of elevated ALT/AST (approximately around 2–5%) in some phase 2 and phase 3 clinical trials (Ellis-Grosse et al., 2005; Oliva et al., 2005; Sacchidanand et al., 2005). The difference might be due to the higher proportion of high-maintenance dose regimes and prolonged duration in this study, compared with these clinical trials. Meanwhile, we noted that the incidence of DILI was lower than the rate of reported-hepatoxicity events in some post-marketing retrospective studies (De Pascale et al., 2014; Chen and Shi 2018; Geng et al., 2018; Xia and Jiang 2020). This could be related to our strict screening criteria and assessment using a standard causality assessment scale, whereas other retrospective studies only considered some abnormal liver enzyme values as adverse events in the liver.

Another key finding was that the most common DILI pattern in this study was cholestatic type, followed by mixed type and hepatocellular type. This finding differed from previous literature involving DILI caused by other medications (Aiso et al., 2019), as tigecycline was the only indicated drug involved in our study. Notably, case series of tetracycline-induced bile duct paucity and prolonged cholestasis with liver histological changes of microvesicular steatosis had been reported previously (Hunt and Washington 1994). Tigecycline-associated DILI presented with cholestatic injury pattern most frequently, which might be explained by its structure derivation from tetracycline, similar with tetracycline in biochemical properties to some extent.

The majority of DILI patients presented with mild liver injury, with the highest proportion of grade 1 (58.2%, 32/55), which may be attributed to the early detection and intervention. Generally, their liver function could be recovered or improved after the withdrawal of the drug. Noticeably, patients in cholestatic injury pattern seemed to be more prone to deteriorate during the course of the disease in our study. Similarly, published papers (Chalasani et al., 2015; Alhaddad et al., 2020; Liu et al., 2020) also revealed that fatal cases and prolonged disease course occurred more frequently among patients with cholestatic injury.

Our study observed that the length of recovery time for all tigecycline-associated DILI patterns ranged from 2 to 66 days after the onset of DILI (Table 1). Previous papers (Andrade et al., 2005; Chalasani et al., 2015) reported that recovery days of the majority of cases exceeded 60 days and the rate of chronic DILI (recovery time after the onset of DILI longer than 180 days) accounted over 10%, regardless of DILI patterns. On the one hand, this could be due to different characteristics between tigecycline and other implicated drugs associated with DILI. On the other hand, the difference in results from other studies could be attributed to the limited follow-up period and undetermined DILI outcome for some cases in the present study. Furthermore, the 26 mentioned patients recovered more quickly than reported mainly due to withdrawal or reduction of tigecycline and provision of hepatoprotective medication timely.

Generally, liver injury patterns could relate to prognosis. Pang et al. (2018) from China observed that patients with cholestatic and mixed injury patterns were more prone to develop chronic liver injury. Previous literature from America (Food and Drug Administration USD of H and HS) revealed that the hepatocellular type was more frequent and predominantly leads to severe DILI. Moreover, other studies showed hepatocellular type and cholestatic liver injury led to poor outcomes and significant mortality (Andrade et al., 2005; Björnsson and Olsson 2005; Chalasani et al., 2008). Regardless of various patterns, it is important to identify DILI and take interventions as early as possible. Our study found that tigecycline-associated DILI seemed not to be the contributory factor for the causes for death within 30 days of treatment after comparing these causes between DILI group and non-DILI group.

Additionally, the present study was the first to report high maintenance dose, prolonged duration, and number of concomitant hepatotoxic drugs as independent risk factors for tigecycline-associated DILI. The effects of off-label use on coagulation function have been noted, but limited data are available to assess effects on liver function (Campany-Herrero et al., 2020). In spite of a meta-analysis showed no significant difference in the incidence of liver injury between the standard-dose group and high-dose group (Zha et al., 2020), the result needed to be interpreted with caution. Because the data used to analyze hepatoxicity came from four single-center, retrospective studies (only 294 patients in total), even the definition of hepatoxicity was not described in two of the studies. To better understand the effects of dose and duration of therapy on tigecycline hepatoxicity, we further performed a stratified analysis. Based on the results, we appealed to clinicians for balancing the relationship between efficacy and safety in caution when choosing a treatment regimen.

The present study has several limitations. First, this was a single-center, retrospective study. The findings relied on the accuracy of medical records and were subject to confound by the propensity to prescribe tigecycline and the bias of the population attended at the department. Second, although all suspected DILI cases were reviewed by consensus of two clinical pharmacists and one clinician, the diagnosis could still be confounded by alternative causes. Third, we acknowledged the limitation of merely calculating a RUCAM score in the DILI assessment. Thus, a prospective study design is recommended in the future to allow for complete data sets and for avoiding possible causality gradings, resulting in a more acceptable and convincing outcome.

Despite as mentioned above, this study also has valuable implications for clinical practice. A strict DILI definition, as well as a standardized, validated causality assessment approach (RUCAM) was applied to objectively and accurately evaluate the occurrence and causality of tigecycline-associated liver injury. Moreover, the study described the main characteristic, management, risk factors and outcome dynamically assessed the evolution of the pattern and severity of tigecycline-associated DILI, which could assist clinicians in managing cases of the elevated liver enzymes after prescribing tigecycline.

## Conclusion

Our study revealed that tigecycline was associated with liver injury, with a slightly higher incidence (5.7%) than the frequency of “frequent” (5%) defined by the Medical Dictionary for Regulatory Activities. In addition, tigecycline-associated DILI is related to high maintenance dose, prolonged duration, and number of concomitant hepatotoxic drugs. Therefore, maintaining diligent monitoring and keen insight is required. Cognizant of this, clinicians should pay particular attention to high maintenance dose and prolonged tigecycline regimen, as well as concomitant use of multiple hepatotoxic drugs.

## Data Availability

The original contributions presented in the study are included in the article/Supplementary files, further inquiries can be directed to the corresponding authors.
